# Gene expression response of the non-target gastropod *Physella acuta* to Fenoxycarb, a juvenile hormone analog pesticide

**DOI:** 10.1038/s41598-023-31201-x

**Published:** 2023-03-10

**Authors:** Patricia Caballero, Marina Prieto-Amador, José-Luis Martínez-Guitarte

**Affiliations:** 1 Grupo de Biología y Toxicología Ambiental, Facultad de Ciencias, Universidad Nacional de Educación a Distancia (UNED), Av de Esparta S/N – Carretera de Las Rozas a El Escorial Km 5, 28232 Las Rozas, Madrid Spain; 2 Grupo de Ecosistemas Bentónicos y Recursos Demersales, COB-Centre Oceanogràfic de Balears (Spanish Institute of Oceanography, CSIC), Moll de Ponent, S/N, 07015 Palma, Illes Balears Spain

**Keywords:** Cell biology, Molecular biology, Environmental sciences

## Abstract

Pesticides are an environmental problem. The search for new pest control methods has focused on compounds with low or no toxic effects in non-target organisms. Analogs of the juvenile hormone (JH) interfere endocrine system of arthropods. However, the lack of effect on non-target species requires confirmation. This article analyzes the impact of Fenoxycarb, an analog of JH, on *Physella acuta*, an aquatic gastropod. For 1 week, animals were exposed to 0.01, 1, and 100 μg/L and the RNA was isolated to analyze the gene expression by retrotranscription and Real-Time PCR. Forty genes related to the endocrine system, the DNA repair mechanisms, the detoxification mechanisms, oxidative stress, the stress response, the nervous system, hypoxia, energy metabolism, the immune system, and apoptosis were analyzed. Three of the genes, *AchE*, *HSP17.9*, and *ApA*, showed responses to the presence of Fenoxycarb at 1 μg/L, with no statistically significant responses in the rest of the genes and at the remaining concentrations. From the results, it can be concluded that Fenoxycarb shows a weak response at the molecular level in *P. acuta* in the tested time and concentrations. However, *Aplysianin-A*, a gene related to immunity, was altered so the long-term effect could be relevant. Therefore, additional research is required to confirm the safety of Fenoxycarb in non-arthropod species in the long term.

## Introduction

The pesticide Fenoxycarb (IUPAC: ethyl [2-(4-phenoxy-phenoxy)ethyl] carbamate, CAS No. 72490-01-8) is a carbamate used to control various insect pests in crops and ornamental cultures^1^. It regulates insect growth by mimicking the juvenile hormone, preventing the insect from reaching maturity^2^. However, it has also been observed in other arthropods^3–5^. At the molecular level, it has been observed that it mediates cell proliferation inhibition and apoptosis in insects^6^ and upregulates the genes involved in juvenile hormone synthesis in spiders^5^. It is considered not harmful to vertebrates and non-target species, affecting insects and other arthropods^5,7–9^. It was reported below the detection level (0.0004 mg/L) 24 to 48 h following aerial application to experimental ponds^10^. Additionally, it has been considered that nominal concentrations of 0.20, 0.80, 3.2, 13, and 50 μg/L brackets predicted environmental levels^11^. Furthermore, Fenoxycarb is considered environmentally safe because of its rapid degradation^10^, with a dissipation time 50 (DT_50_) of 4.13 days in the water column and 15 days in sediment (PPDB database: http://sitem.herts.ac.uk/aeru/ppdb/en/Reports/304.htm)^1^. It has a low drift to adjacent lands from fields where it is applied^12^, although it may end up in surface waters due to spray drift, runoff, or drainage^13^. It has been banned in the EU but is in use in the UK, USA, and Australia. European Food Safety Authority considered it with no areas of concern in the areas of mammalian toxicology and consumer risk assessment for using fenoxycarb in apples and pears^14^. However, while a low risk was assessed for birds and mammals, soil non-target macro and micro-organisms, and terrestrial non-target plants, a high risk was identified for aquatic organisms with a data gap in the risk to aquatic invertebrates considering the mode of action and the most sensitive growth stages^14^. Although it is considered with low toxicity upon oral, dermal or inhalation exposure, it has been proposed to be classified as a chemical with limited evidence of a carcinogenic effect because it can produce lung and liver tumors in mice by inducing peroxisome proliferation, a mechanism less sensitive in humans^14^. Because of it, fenoxycarb is labeled as very toxic for aquatic life and suspected of causing cancer.

In vertebrates, no effect on reproduction in sheep was observed^15^, but it has been described that in cultured rat cortical neurons exposed for one week, Fenoxycarb considerably decreased ATP levels, mitochondrial membrane potential, and glucose consumption^16^. Furthermore, it inhibits rat brain acetylcholinesterase and nicotinic acetylcholine receptors expressed in *Xenopus laevis* oocytes^17^. On the other hand, some reports show that it can negatively affect egg production and the hatching rate in the collembola *Yuukianura szeptyckii*^18^, although previous studies on *Folsomia candida* did not show such an effect^19^. Similarly, adverse effects such as inhibition of molting and body length growth were observed when the shrimp *Neocaridina davidi* was exposed for two weeks to concentration as low as 10 μg/L^3^, while the crab *Rhithropanopeus harrisii* experienced delayed metamorphosis at 48 μg/L of Fenoxycarb^20^. Taking together these results, additional studies are required to know the impact of this pesticide on ecosystems.

There is a lack of information on the effects of Fenoxycarb on non-arthropod freshwater invertebrates, but it is expected to be harmless to them. However, the paucity of knowledge of the physiology of invertebrates, especially regarding the endocrine system, requires confirmation of this extreme. This work aims to test the toxicity of Fenoxycarb at the transcription level, in the freshwater gastropod *Physella acuta* (Draparnaud, 1805) by exposing the animals for 1 week and analyzing the transcription profile with an array covering different relevant cellular pathways.

The freshwater snail *Physella acuta*, also known as *Physa acuta*, is a hermaphroditic and cosmopolitan species. It lives in lakes and ponds and lays its eggs in an egg mass that requires around two weeks to develop. The hatched juveniles grow for two months until they reach the adult stage, mate, and lay eggs. The species is easily cultured in the laboratory, so it is used in toxicity studies as representative of the gastropods^21,22^. Recently, we designed an array to study the response to toxicants at the gene expression level in this species^23^. It included 34 genes and 4 reference genes. In addition, we extended it to 40 target genes, including some of them to analyze the alterations at the transcriptional activity level in several cellular processes. The sequence of nine genes is described for the first time for this species and extends the number of genes that can be used as biomarkers. The sequences code for proteins related to the endocrine system (galanin receptor type 2 [GalR2], estrogen-related receptor [ERR], membrane progestin receptor-beta [MPR], estradiol 17-beta-dehydrogenase 8 [Hsd17b8], and retinoic acid receptor [RXR]), DNA repair (poly-ADP-ribose polymerase I [PARP1], DNA repair protein XRCC3 [XRCC3], nuclear factor of kappa light polypeptide gene enhancer in B-cells inhibitor, alpha [IkBa]), and stress response (heat shock protein 70 B2-like [HSP70 B2]). Overall, the array allows analysis of alterations in the endocrine system, detoxification mechanisms, DNA repair, the nervous system, apoptosis, oxidative stress, stress, epigenetics, the immune system, energy metabolism, and lipid transport. In this way, the array shows changes in the main mechanisms involved in the response to stress and detoxification but also the response of processes involved in long-term effects, such as epigenetic modification mechanisms and DNA repair, that would be activated in case of any genotoxic effect of the compound.

Safety at the environmental level is a concern of all the products used as pesticides. The search for new pesticides with a reduced impact in non-target species demands testing in these species because some of them can have low impact. However, the lack of knowledge about the physiology of invertebrates requires experimental work to confirm it. It will also provide additional information about the physiology of the invertebrates, decreasing the gap with the vertebrates and favoring the use of invertebrates as alternative methods that reduce the use of vertebrates in the test of toxicity.

## Results

### Identification of sequences

**Figure 1:**
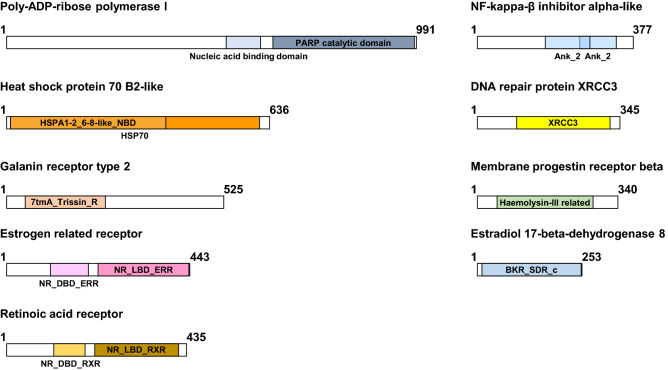
HYPERLINK "sps:id::fig1||locator::gr1||MediaObject::0"Structure and conserved domains of the *Physella acuta* proteins code by the sequences identified. The characteristic motifs of each protein are shown. The domains have been defined according to the Conserved Domains Database (CCD) functional classification of proteins. The size is indicated by the numbers.

Nine sequences were identified that code for different proteins related to the endocrine system (*Hsd17b8*, *ERR*, *GalR2*, *MPR*, and *RXR*), the stress response (*HSP70B2*), and DNA repair mechanisms (*XRCC3*, *IkBa*, and *PARP1*). The size of the sequence and the ORF size are shown in Table 1. Furthermore, the identity and similarity at the amino acid level with the indicated protein are shown. The database comparison showed homology with proteins from other mollusks, mainly gastropods, except for MPR, which was homologous to a protein of bivalve origin. The degree of homology was high except in the case of the GalR2 and the MPR, while ERR and HSP70 B2 showed more than 90% identity. Figure 1 shows the scheme of the proteins with the different motifs that characterize them. All of them showed the characteristic domains associated with those proteins, so it can be concluded that the isolated sequences correspond to the genes coding those proteins.

**Table 1:** Information of the sequences described for first time.

Contig/acc. number	Gene	Size bp (DNA)	Size aa (prot)	Homology	Identity (%)	Similarity (%)
Contig11036	DNA repair protein XRCC3	1273	345	PREDICTED: DNA repair protein XRCC3-like isoform X1—*Biomphalaria glabrata* XP_013089306	58	72
OK474809	Estradiol 17-beta-dehydrogenase 8	1055	253	17 beta-hydroxysteroid dehydrogenase 8—*Lymnaea stagnalis* QNG40045	78	90
Contig1634	Estrogen related receptor	3125	443	PREDICTED: steroid hormone receptor ERR2-like isoform X3—*Biomphalaria glabrata* XP_013080351	91	96
OK474807	Galanin receptor type 2	2066	525	PREDICTED: galanin receptor type 2-like—*Biomphalaria glabrata* XP_013071428	38	55
OK474812	Heat shock protein 70 B2-like	2486	636	PREDICTED: heat shock protein 70 B2-like—*Biomphalaria glabrata* XP_013072147	91	96
Contig8155	NF-kappa-B inhibitor alpha	1611	377	PREDICTED: NF-kappa-B inhibitor alpha-like—*Biomphalaria glabrata* XP_013067082	57	74
OK474808	Membrane progestin receptor beta	2308	340	membrane progestin receptor beta-like—*Mizuhopecten yessoensis* XP_021341591	39	58
OK474811	Poly-ADP-ribose polymerase I	3162	991	poly-(ADP-ribose) polymerase I—*Aplysia californica* NP_001191521	77	87
OK474810	Retinoic acid receptor RXR	3551	435	Retinoic acid receptor RXR—*Lymnaea stagnalis* Q5I7G2	96	97

DNA and protein size, homology, identity, and similarity are indicated. The contig number or accession number are indicated.

### Gene expression profile in response to Fenoxycarb exposure

**Figure 2:**
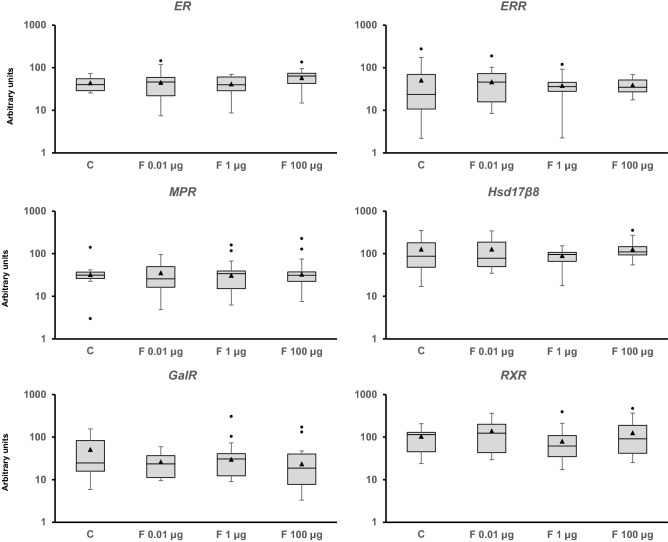
Transcript levels of endocrine-related sequences (*estrogen receptor* [*ER*], *estrogen-related receptor* [*EER*], *membrane progestin receptor beta* [*MPR*], *estradiol 17-beta-dehydrogenase 8* [*Hsd17b8*], *galanin receptor 2* [*GalR2*], and *retinoic acid receptor* [*RXR*]) in *Physella acuta* adults after in vivo exposure to Fenoxycarb for seven days at 19 °C. Transcriptional activity was quantified by RT-PCR using *ribosomal protein L10* (*rpL10*), *actin* (*Act*), *6-phosphofructo-2-kinase/fructose-2,6-biphosphatase 2* (*PFKFB2*), and *glyceraldehyde-3-phosphate dehydrogenase* (*GAPDH*) as reference genes. The comparison was performed with the solvent-exposed controls. Whisker boxes are shown. Each box corresponds to 12 individuals. The horizontal line within the box indicates the median, and the 25th and 75th percentiles are indicated by the boundaries of the box. The highest and lowest results are represented by the whiskers. The small triangle inside the box denotes the mean, and the outliers are shown (circles). No significant differences to control were observed in those genes (p < 0.05).

**Figure 3:**
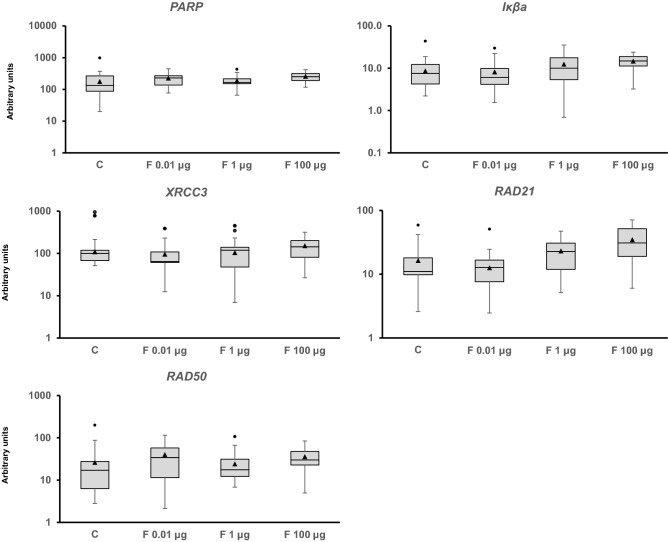
Transcriptional activity of genes related to DNA repair. The mRNA levels of *poly(ADP-Ribose) polymerase I* (*PARP1*), *NFKB inhibitor Iκβ* (*IkBa*), *X-ray repair cross complementing 3* (*XRCC3*), *RAD21 Cohesin Complex Component* (*RAD21*), and *RAD50 Double Strand Break Repair Protein* (*RAD50*) in *Physella acuta* adults after in vivo exposure to Fenoxycarb for seven days at 19 °C are shown. RT-PCR was used to quantify the mRNA levels and *ribosomal protein L10* (*rpL10*), *actin* (*Act*), *6-phosphofructo-2-kinase/fructose-2,6-biphosphatase 2* (*PFKFB2*), and *glyceraldehyde-3-phosphate dehydrogenase* (*GAPDH*) were used as reference genes. The comparison was performed with the solvent-exposed controls. Whisker boxes are shown. Each box corresponds to 12 individuals. The median is indicated by the horizontal line within the box, and the 25th and 75th percentiles are indicated by the boundaries of the box. The highest and lowest results are represented by the whiskers. The small triangle inside the box denotes the mean, and the outliers are shown (circles). No significant differences were observed relative to the control (p < 0.05).

**Figure 4:**
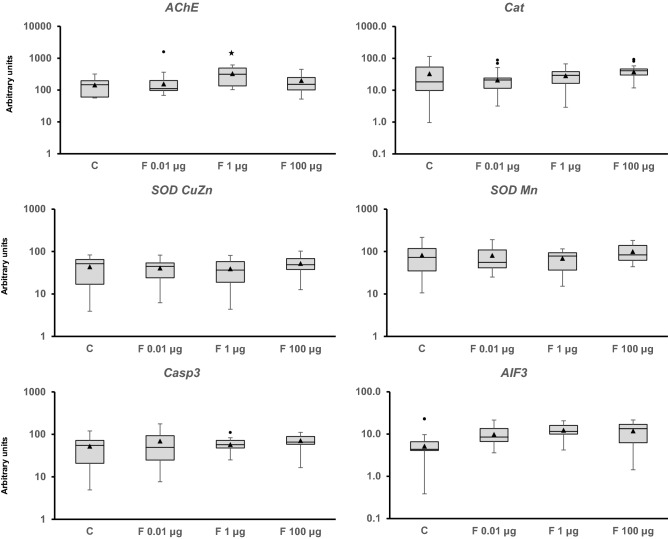
Transcriptional activity of genes related to the nervous system (*acetylcholinesterase* [*AChE*]), oxidative stress (*catalase* [*Cat*], copper-zinc superoxide dismutase [*SOD CuZn*], and *manganese superoxide dismutase* [*SOD Mn*]), and apoptosis (*caspase 3* [*Casp3*] and *apoptosis inducing factor 3* [*AIF3*]). Snails were exposed to Fenoxycarb for seven days at 19 °C. Quantification by RT-PCR was performed using *ribosomal protein L10* (*rpL10*), *actin* (*Act*), *6-phosphofructo-2-kinase/fructose-2,6-biphosphatase 2* (*PFKFB2*), and *glyceraldehyde-3-phosphate dehydrogenase* (*GAPDH*) as reference genes. The comparison was performed with the solvent-exposed controls. Whisker boxes are shown. Each box corresponds to 12 individuals. The median is indicated by the horizontal line within the box, and the 25th and 75th percentiles are indicated by the boundaries of the box. The highest and lowest results are represented by the whiskers. The small triangle inside the box denotes the mean, and the outliers are shown (circles). Significant difference relative to the control (asterisk) is indicated (p < 0.05).

**Figure 5:**
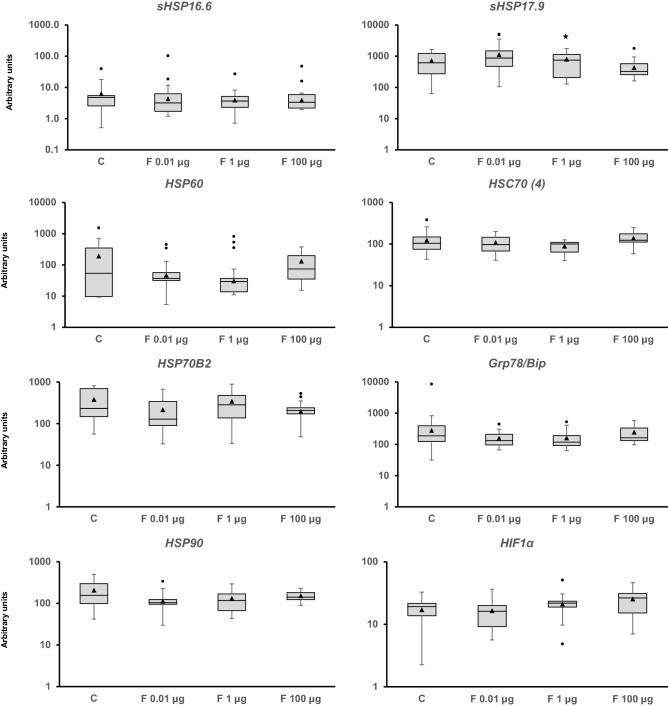
Transcriptional activity of stress (*small heat shock protein 16.6* [*sHSP16.6*], *small heat shock protein 17.9* [*sHSP17.9*], heat shock protein 60 [*HSP60*], *heat shock cognate 70 4* [*HSC70 (4)*], *heat shock protein 70 B2-like* [*HSP70B2*], *glucose regulated protein 78/binding immunoglobulin protein* [*Grp78/BiP*], and *heat shock protein 83* [*HSP83*]) and *hypoxia-inducible factor-1 alpha* [*HIF1α*]) genes in adult snails. The animals were exposed for one week to Fenoxycarb at 19 °C. Transcriptional activity was quantified by RT-PCR using *ribosomal protein L10* (*rpL10*), *actin* (*Act*), *6-phosphofructo-2-kinase/fructose-2,6-biphosphatase 2* (*PFKFB2*), and *glyceraldehyde-3-phosphate dehydrogenase* (*GAPDH*) as reference genes. The comparison was performed with the solvent-exposed controls. Whisker boxes are shown. Each box corresponds to 12 individuals. The median is indicated by the horizontal line within the box, and the 25th and 75th percentiles are indicated by the boundaries of the box. The highest and lowest results are represented by the whiskers. The small triangle inside the box denotes the mean, and the outliers are shown (circles). Significant difference relative to the control (asterisk) is indicated (p < 0.05).

**Figure 6:**
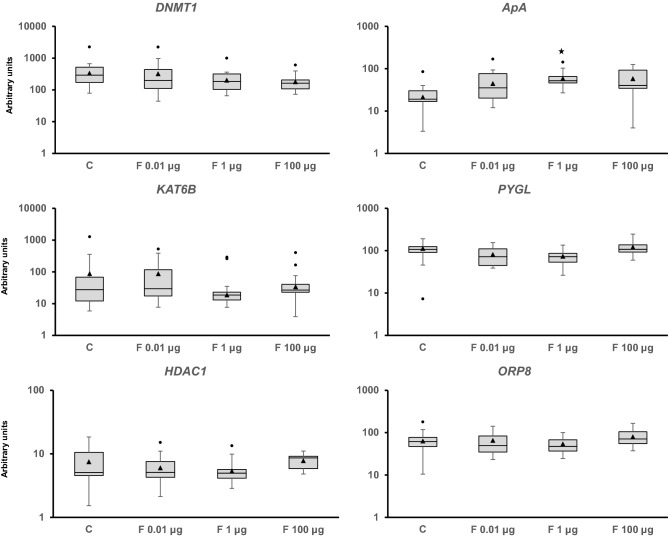
Transcriptional activity of genes related to epigenetic modulation (*DNA methylase I* [*DNMT1*], *Lysine Acetyltransferase 6B* [*KAT6B*], and *histone deacetylase 1* [*Hda1*]), immunity (*aplysianin-A* [*ApA*]), and energy metabolism (*glycogen phosphorylase L* [*PYGL*] and *oxysterol binding protein like 8* [*OSBPL8*]) in *Physella acuta* adults after in vivo exposure to Fenoxycarb for seven days at 19 °C. Transcriptional activity was quantified by RT-PCR using *ribosomal protein L10* (*rpL10*), *actin* (*Act*), *6-phosphofructo-2-kinase/fructose-2,6-biphosphatase 2* (*PFKFB2*), and *glyceraldehyde-3-phosphate dehydrogenase* (*GAPDH*) as reference genes. The comparison was performed with the solvent-exposed controls. Whisker boxes are shown. Each box corresponds to 12 individuals. The median is indicated by the horizontal line within the box, and the 25th and 75th percentiles are indicated by the boundaries of the box. The highest and lowest results are represented by the whiskers. The small triangle inside the box denotes the mean, and the outliers are shown (circles). Significant difference relative to the controls (asterisk) is indicated (p < 0.05).

Adult snails were exposed to 0.01, 1, and 100 µg/L of Fenoxycarb for seven days to assess the mid-term response of the genes analyzed (Figs. 2, 3, 4, 5,6, S1 and S2). Fenoxycarb is an analog of juvenile hormone that is not expected to affect non-arthropods. There was no statistically significant response in those genes related to the endocrine system (Fig. 2), DNA repair mechanisms (Fig. 3), oxidative stress (Fig. 4), apoptosis (Fig. 4), phase I (Fig. S1), phase II (Fig. S2), and phase III (Fig. S2) of detoxification, most stress proteins (Fig. 5), hypoxia (Fig. 5), epigenetic regulation (Fig. 6), and energy metabolism (Fig. 6). Only three genes were modified at 1 µg/L: *acetylcholinesterase* (*AChE*) (chi square = − 16.144, p = 0.029; Fig. 4), *heat shock protein 17.9* (*HSP 17.9*) (chi square = − 15.553, p = 0.039; Fig. 5), and *aplysianin A* (*ApA*) (chi square = − 20.333, p = 0.002; Fig. 6). Therefore, the two genes analyzed in relation to the nervous system (*AChE*) and immunity (*ApA*) showed some alteration, suggesting some mid-term effects on the physiology of *P. acuta* could occur such as feed searching behaviour or susceptibility to bacterial infections.

## Discussion

Advances in toxicological studies demand the extension of toxicity tests to additional levels beyond traditional endpoints such as survival, reproduction, or development. Nowadays, a molecular approach to toxicity evaluation is frequently taken, and it requires additional putative biomarkers that assess the modulation of different cellular processes and physiological mechanisms^24–26^. In this sense, adding new genes to the battery of biomarkers extends the number of processes studied and the levels of response, depending on the pathway analyzed. Therefore, the description of new genes is a step toward extending the value of *P. acuta* in toxicological studies. Here we have described nine new sequences that code for different hormone receptors, an enzyme involved in regulating the concentration of active estrogens and androgens (Hsd17b8), one stress protein, and three proteins involved in the DNA repair. These genes can improve the analysis of different processes related to endocrine disruption, genotoxicity, and development. Furthermore, all of them can help us to understand the response on different levels of organization, from molecular to ecological, providing insights into the mechanisms of the toxicant and the responses of the organisms to maintain homeostasis in the face of a changing environment. On the other hand, these putative biomarkers open new ways to assess toxicity prior to its observation at the individual level, preventing irreversible damage that affects the population. Thus, as in clinical practice, new tools are required to better identify molecular events and obtain an earlier diagnosis that will help to detect pollution before it causes irreversible damaging effects on ecosystems.

For a long time, the search for pesticides with either low or no impact on non-target species has been a key agricultural aim. Analogs of the juvenile hormone have been one such pesticide since they mimic one specific hormone of arthropods, drastically reducing the risk to other species^27^. It is known that Fenoxycarb affects the development and different cell processes in insects, arachnids, and crustaceans^8,13,18^. However, the current poor knowledge of invertebrate physiology makes it necessary to test them in non-target species to ensure their low impact. The first element to consider is the fact that the response is observed at 1 μg/L of Fenoxycarb, which is the intermediate concentration used. It is very low compared to those that have an effect on insects and crustaceans^3,20,28^. The lack of response at higher concentrations could be due to an earlier response, recovering the normal condition to the time of the analysis by the action of detoxification mechanisms. At the lower concentration, the lack of observed response could be due to the amount of toxicant being below the threshold concentration needed to trigger an effect. Another possibility could be that more time is necessary to reach the threshold concentration. Finally, it cannot be discarded either a putative U-shape response. Additional research employing different response times would allow elucidation of the cellular processes affected and the concentration-dependent consequences.

In this work, we have tested the response at the gene expression level of the analog of juvenile hormone, Fenoxycarb and observed a response that, although weak, demands additional studies to ensure the lack of toxicity in non-arthropods. As expected, no effect was observed in genes related to DNA repair mechanisms or the stress response. There are no data in the literature on studies of these genes, even in insects. A similar situation happens with energy metabolism, although there are some reports about the impact of fenoxycarb in lipids and carbohydrates of crustaceans^3,4,29^. As far as we know, there is no previous report analyzing the response of detoxification mechanisms in the presence of Fenoxycarb. In *P. acuta* there is no change in the genes analyzed involved in phase I, phase II, and phase III of detoxification, suggesting that other proteins different from those analyzed here are responsible for the biotransformation of this chemical. In contrast to our results, with no changes in genes related to epigenetic regulation, it has been described that exposure for three days at 50 µg/L of Fenoxycarb can upregulate histone deacetylase in the water flea *Moina macrocopa*^4^. This could reflect differential sensitivity to the compound but also the fact that the epigenetic changes in the water flea are related to mimicking of the juvenile hormone effects.

The effect on the *AChE* suggests some impact on the nervous system. As stated in the introduction, inhibition on rat brain acetylcholinesterase activity and nicotinic receptors have been observed^17^, suggesting that Fenoxycarb can have nervous effects on non-target organisms. Studies in nicotinic receptors showed that the mechanism of Fenoxycarb was noncompetitive^30^. Although Fenoxycarb has been used as an analog of juvenile hormone, it seems to also have some effect as the rest of carbamates by affecting the nervous system. The response observed in *P. acuta* supports a nervous effect and suggests that it could affect the ability of the snail to survive by altering the central nervous system. The increase observed in the transcription could be reflecting and attempt to compensate the inhibition on enzyme activity. Additional studies would help elucidate the putative effect on the snail’s behavior or ability to respond to situations involving the nervous system. In any case, it is a fact to consider in the impact that Fenoxycarb can have in non-target species in the mid- and long-term.

On the other hand, the modulation of *sHSP17.9* suggests some effect, but to determine the real impact on the cell is a complex matter. Small heat shock proteins are diverse proteins involved in the stress response and related to multiple cellular processes, including neural functions^31^. In this sense, it is tempting to speculate that *sHSP17.9* could encode some sHSP involved in neural physiology but the difficulties to establish the homology demand caution. Additional research will provide more information and could help to define the role of this protein in the cell. As biomarkers, the fact that sHSPs share the alpha-crystallin domain makes it easy to identify them. However, their high diversity in the N- and C-terminal regions complicate the identification of homologies between species. Consequently, a deeper study of this protein family is required to determine their roles in cell metabolism and to establish functional homologies between them. In any case, the alteration observed suggests that Fenoxycarb has some effect in the mid-term in *P. acuta*, raising the possibility that it causes some reduction in the wellness of the snail.

Aplysianin-A is a protein involved in the immune response by acting as an antibacterial. This antibacterial glycoprotein inhibits both gram-positive and gram-negative bacteria in *Aplysia kurodai*^32^. The alteration in transcriptional activity can produce a modulation of the response to bacterial infections, making *P. acuta* more sensitive to them. An excess in the response or in an inadequate moment could affect the ability of the organism to respond, selecting those bacteria which are not sensitive to ApA, making the animal more sensitive to infection. The impact observed in *P. acuta* suggests a putative alteration in immunity, being the first time that this possibility is suggested for Fenoxycarb. Additional studies involving more immune related genes are needed to confirm it and determine the effect in the long-term survival of the population.

The present evidence suggests a low impact on the environment of Fenoxycarb. However, the results observed in the non-target species *P. acuta* require an extended analysis because the exposure can reflect a long-term impact. Although the data at the molecular level suggest a weak effect in the short-term, additional analyses testing other parameters will provide additional evidence. To ensure the harmless of the Fenoxycarb, it should be analyzed in several non-target species covering different groups of invertebrates and testing short and long-term effects at molecular, cellular, and organism levels.

## Materials and methods

### Chemicals

Fenoxycarb was purchased from Sigma-Aldrich (Germany). In addition, TRIzol and Moloney Murine Leukemia Virus (M-MLV) enzyme were obtained from Invitrogen (Germany), oligonucleotide poly dT_18_ primer and gene-specific primers were supplied by Macrogen (Korea), RNase-free DNase was purchased from Sigma (Germany), DNA polymerase and dNTPs were obtained from Biotools (Spain), and EvaGreen was purchased from Biotium (USA).

### Animals

The populations of *Physella acuta* were grown in the laboratory for numerous generations. They were established from animals provided by Dr. Sánchez-Argüello (Instituto Nacional de Investigación y Tecnología Agraria y Alimentaria, Spain). The gelatinous egg masses were collected and allocated in 500 mL glass vessels with 250 mL of culture medium (2 mM CaCl_2_, 0.5 mM MgSO_4_, 0.77 mM NaHCO_3_, and 0.08 mM KCl). The medium was changed twice per week, and the animals were fed twice per week with a mixture of Shrimps Natural (Sera) and Micron Nature (Sera). The cultures were maintained at 18 ± 1 °C under a 16 h light:8 h dark cycle.

### Treatment

Fenoxycarb was diluted in acetone for a final stock of 100 mg/mL. Fresh stock was diluted to final concentrations of 0, 0.01, 1, and 100 μg/L (0, 0.031, 3.31, and 331.8 nM respectively). The concentrations were selected as predicted environmentally relevant concentrations following Hosmer et al.^11^. As the present work aimed to evaluate mid- and long-term effects, seven days were selected because gene expression at this time could be considered stabilized in comparison to the acute response at hours of treatment (up to 24 h). The exposure was performed in glass vessels with 300 mL of culture medium. In each vessel were placed six two-month-old adult snails (0.091 ± 0.01 g and 0.79 ± 0.08 cm). The control was performed with the solvent at the same concentration of treatments, 1:10,000. The medium was renewed after three days, containing the same concentration of Fenoxycarb in each case, and the animals were fed with 18 mg of the food mixture (3 mg/animal). In each experiment, three of the snails were recovered for mRNA analysis per concentration. The animals were individually frozen directly in a microtube on dry ice. Four experiments were performed, so twelve biological replicates (n = 12) were used for each concentration. The treatments were at 18 °C and 16:8 light: dark cycle. There was no mortality during the experiments.

### Sequence identification and primer design

As previously stated, most array genes have been described in another article^23^. They are summarized in Table S1. Nine genes are described here for the first time. The sequences were isolated from a transcriptome obtained in the laboratory^33^ and the sequences published by^34^, which are available on the web (http://kimura.univ-montp2.fr/PopPhyl/index.php?section=data#dataset_0, PopPhyl Contigs, Physa_acuta#solo200.fas). The sequences obtained from the transcriptome and Romiguier et al.^34^ were identified by blasting them to the database using the txblast tool, a non-redundant database, and an e-value threshold of 1e−3. The search was performed with OmicsBox [OmicsBox—Bioinformatics made easy (Version 2.0.29). BioBam Bioinformatics. March 3, 2019. https://www.biobam.com/omicsbox]. The identified sequences were translated with Snapgene software (GSL Biotech LLC, USA), and the protein was compared with the GenBank protein database to confirm the identity of the gene. *ERR*, *IkBa*, and *XRCC3* were identified from sequences published by Romiguier et al.^34^ (Table 1), while *GalR2*, *MPR*, *Hsd17b8*, *RXR*, *PARP1*, and *HSP70 B2* were obtained from the transcriptome. The accession numbers of transcriptome sequences are OK474807 (*GalR2*), OK474808 (*MPR*), OK474809 (*Hsd17b8*), OK474810 (*RXR*), OK474811 (*PARP1*), and OK474812 (*HSP70B2*) (Table 1).

Primers were designed using Primer-Blast (Ye et al. 2012)^35^. The amplicon size was 100–200 bp, and the optimal melting temperature was 58 °C. All primer pairs were tested by polymerase chain reaction (PCR) in a C1000 thermocycler (Bio-Rad, USA) with DNA AmpliTools Green Master Mix (Biotools, Spain). The single band was confirmed by gel electrophoresis (1.5% agarose gel). The PCR program was the same as that used for real-time PCR (RT-PCR).

### RNA extraction and retrotranscription

Frozen adults were used to perform the RNA extraction. Each animal was processed individually. RNA extraction using TRIzol was performed by following the manufacturer’s indications. Briefly, the frozen adult was homogenized immediately and stored at − 80 °C. For the extraction, the samples were thawed at room temperature and chloroform was added to the sample and mixed. They were incubated for three min at room temperature. Afterward, the sample was centrifuged for 10 min at 4 °C, and the upper phase recovered. The RNA was precipitated with 0.7 volumes of isopropanol and washed with 75% ethanol. The RNA was resuspended in diethylpyrocarbonate-treated water and incubated for 45 min with RNAse-free DNAse (Roche, Germany). A phenol: chloroform treatment was performed with Phase-Lock tubes (5 prime, USA) to remove the DNAse. The RNA was precipitated with isopropanol and resuspended in 100 µL of DEPC-treated water.

Retrotranscription was performed with MMLV (Invitrogen, Germany) in a final volume of 40 µL with 10 µg of RNA following the manufacturer’s indications. The primer used was a poly dT_18_. The retrotranscribed sample was maintained at − 20 °C until use.

### Real-time PCR

RT-PCR was performed using a 96-well plate with 40 target genes and 4 reference genes, so each gene was analyzed in duplicate. The primers sequences can be found in the supplementary material (Table S2). First, the efficiency of each primer pair was established by amplifying the sequence by PCR employing the same conditions as in RT- PCR. Then, a 1:25,000 dilution was performed by mixing the PCRs of array genes, mimicking the variety of sequences in an RNA extraction. Finally, a five-dilution series was used to obtain the efficiency curve with the same program used for RT-PCR (see below). Efficiency is listed in Table S2.

First, the primers were added to each well (250 nM each). Then, a master mix with the cDNA (8 µL per plate), Evagreen (0.5 ×), dNTPs (0.2 mM), 1 × buffer, and 2.5 mM MgCl_2_ was prepared, and 10 µL were added to each well. Two technical replicates were performed for each sample. The program used was an initial denaturation at 95 °C for 2 min and then 95 °C for 15 s, 58 °C for 30 s, and 72 °C for 15 s, repeated 39 times. After that, a melting curve from 60 to 85 °C was constructed with 0.5 °C steps to confirm the presence of a single product. To establish the cycle threshold, the regression option was used in the Maestro software (BioRad). The Ct was the value used for subsequent data analysis by the 2^−ΔΔCt^ method^36^. The mean Ct of the control samples was used as a control reference for all the control and treated.

### Statistics

The statistical analysis was performed in SPSS 25 (IBM, USA). The data showed no normal distribution when the Shapiro–Wilk test was performed; therefore, the non-parametric Kruskal–Wallis test was used for data analysis. The level of significance was set at p ≤ 0.05. The number of samples per condition was n = 12. Statistically significant changes relative to the control were considered as alterations.

## Supplementary Information

Supplementary Information.

## Data Availability

The sequences analysed during the current study are available in the GenBank (https://www.ncbi.nlm.nih.gov/nucleotide/) with accession numbers OK474807, OK474808, OK474809, OK474810, OK474811, and OK474812 (released in November 1st, 2022). The Romiguier et al.^34^ contigs can be found in PopPhyl website (https://kimura.univ-montp2.fr/PopPhyl/index.php?section=home), in dataset section (https://kimura.univ-montp2.fr/PopPhyl/index.php?section=data).
