# Supplementary material for: Gene expression response of the non-target gastropod Physella acuta to Fenoxycarb, a juvenile hormone analog pesticide
Source: Sci Rep. 2023 Mar 10;13:4031. doi: 10.1038/s41598-023-31201-x (PMC10006217; doi:10.1038/s41598-023-31201-x)
Supplement: Supplementary file 1 — Supplementary Information. [file 41598_2023_31201_MOESM1_ESM.pdf]

Gene expression response of the non-target gastropod *Physella acuta* to Fenoxycarb, a juvenile hormone analog pesticide

Patricia Caballero, Marina Prieto-Amador, José-Luis Martínez-Guitarte

Grupo de Biología y Toxicología Ambiental. Facultad de Ciencias. Universidad Nacional de Educación a Distancia (UNED)

Table S1. Summary of the genes used in the array and the associated cell processes

<table>
<tr><th>Process</th><th>Gene</th><th>Process</th><th>Gene</th><th>Process</th><th>Gene</th><th>Process</th><th>Gene</th></tr>
<tr><td rowspan="5">Endocrine system</td><td rowspan="5">ER
ERR
MPR
Hsd17b8
GalR2
RXR</td><td>Nervous system</td><td>AChE</td><td rowspan="2">Epigenetics</td><td rowspan="2">DNMT1
KAT6B
Hda1</td><td rowspan="2">Oxidative stress</td><td rowspan="2">Cat
SOD CuZn
SOD Mn</td></tr>
<tr><td>Apoptosis</td><td>Casp3
AIF3</td></tr>
<tr><td rowspan="6">Detoxification</td><td rowspan="6">Cyp 2U1
Cyp 3A7
Cyp 4F22
Cyp 72A15
GST K1
GST M1
GST O1
GST T2
Mrp1/ABCC1</td><td rowspan="5">Stress</td><td rowspan="5">sHSP 16.6
sHSP 17.9
Hsp60
Hsc70 4
Hsp70 B2
Grp78/Bip
Hsp83</td><td>Glycogen</td><td>PYGL</td></tr>
<tr><td>Lipids</td><td>OSBPL8</td></tr>
<tr><td>Antibacterial</td><td>ApA</td></tr>
<tr><td rowspan="3">Repairing DNA</td><td rowspan="3">PARP I
IkBa
XRCC3
rad21
rad50</td><td rowspan="3">Reference</td><td rowspan="3">Act
PHFK
GAPDH
rpL10</td></tr>
<tr></tr>
<tr><td>Hypoxia</td><td>HIF1a</td></tr>
</table>

Table S2. Primer sequence and efficiency of the primer set for each gene

Gen	Primer	Secuencia	Efficiency
ER	F	CTATGCCAGGTGTGTAATGAC	79.6
	R	CGAAGCCGACATGCTTGACA	
ERR	F	GATGCTCTCTCAGACTGTGTC	89.5
	R	AGCATTTCCAGGAAGAGC	
MPR	F	CTGGCCAATGTTGGGTTTCG	99.9
	R	CGCCTGCGTAGTCTATCTGG	
Hsd17b8	F	AGACAAACTACGCTGCGTCT	103
	R	TGGCAGGATGGCATTCACTC	
GalR2	F	TCGGATGCGGTGTTTGAGAA	101.6
	R	TCCGTGTCGTAGGAGGAGTT	
RXR	F	ACCAGTGGCGCCAATAATGA	80.1
	R	TGAAGAGCTGCTTGTCTGCT	
PARP 1	F	CATGCTGAGACACACAACACA	98.4
	R	TGTTGTACGAGACCCATGCC	
IkBa	F	CAAGAGTGGACGCTCTGTCT	103

	R	CCACTGCCAGCAGAATAGGT	
XRCC3	F<br>R	TCTGGTGAAAGTGCATGTGGA<br>TGGAGACGCTTGCTTGGAAA	104.6
rad21	F<br>R	CCGGCCAATGTCTGATGACT<br>GCAATTGCTTGCTGGCATCT	96.9
rad50	F<br>R	AGGCAAGGAGGAGCTACAAC<br>TTCAGCCAATGCTAAGCGGA	98.7
AChE	F<br>R	AGTGTCCCGTCGTGGATTTC<br>CACGACCTCGATCTCGTAGC	89.8
Casp3	F<br>R	GTCTGTGTAATTCTCACCCATG<br>AGTTCAGTGCCTCTGCAAGC	107.2
AIF3	F<br>R	ACCACAAGATGCCAACGCTA<br>ACTGGCAGCCTTATCAGCAA	102
Cyp 2U1	F<br>R	GTGCATCCTCTACGCGATCA<br>GGCTAGTTTGGGCCTGTCTT	102.1
Cyp 3A7	F<br>R	ACGGCTTGGCCTCTCAATAC<br>CGGTTTCTTTCTCGGCGTTC	84.8
Cyp 4F22	F<br>R	AGCAGAAAAAGCTCAGCCCT<br>CTTGGTTTTGGCAGCCAGTC	87.2
Cyp 72A15	F<br>R	AGGGAAGTGGCTTGAGTGAC<br>GGTGCTCAGCCAGCATAAGA	91.9
GST K1	F<br>R	TGAGCAGAGTAGTTTGGCTGC<br>ATGCCCCTAATTCTGTGGCT	96.7
GST M1	F<br>R	ATTGGGCCATTAGAGGGCTT<br>GTTGGACCATCTCCTTGCAC	93.1
GST O1	F<br>R	CCACCTGGCAACTTGGTTTG<br>GCTTGCCAGATGCGTAAGAC	92.8
GST T2	F<br>R	TCGATCTTCTATCGCAGCCG<br>TTCTGAGCGCAACAGGTTTG	86
Mrp1/ABCC1	F<br>R	CAGGGGCAGGTAAGTCATCC<br>AGTGAGCCTTGATCGCACAT	94.5
DNMT1	F<br>R	GACGCCATGTCCGATTTACCT<br>TCATCCGCGCTGCCACCAG	93.3
KAT6B	F<br>R	CTTCCATGGGGATGACGAGG<br>AAGCTTTGAACGTTTGCCCC	95.1
Hda1	F<br>R	CCCATCAAACATGGCCAACC<br>GTGCATGTGGCAACATTCTGA	90.8
sHSP 16.6	F<br>R	GCATGAGGAGAAGCAAGACA<br>CAGTACACCATGGGCATTCA	96.4
sHSP 17.9	F<br>R	TTCACGCGTTGGTGAATCAG<br>TTAGCAGCTACAGTCAGCGT	102.5
Hsp60	F	GGCACTAAGGGTTCCTGCTC	82

	R	ATCCGATATCGCCAGCAGAG	
Hsc70 4	F	TGGTGTGCCCCAGATTGAAG	107.8
	R	TCCTCTTTGGACAGACGACC	
Hsp70 B2	F	CTGGAGGCGTTATGACTG	95.2
	R	AGGTGAAATCGACCCAAG	
Grp78/Bip	F	TGGTGGCTCAACCCGTATTC	96.8
	R	TCCCCACTCAAAACACCAGC	
Hsp83	F	GTTTGTGTCACTAAAGAAGGCC	91.8
	R	TGTCACTAGCCTATTTGATACAACC	
HIF1a	F	AGGATAGATGCTGGCACACC	81.4
	R	CATAGACACGGTCCTCCCCT	
Cat	F	CCCAGTCAGTGGTGATGTCC	98.8
	R	TTCAGGTGCCCGACAATGTT	
SOD CuZn	F	AGAAAGCTGGTGCTGCAACTA	104.9
	R	AGGATTAAAGTGGCCTCCAGC	
SOD Mn	F	TCGAATTGCTACCTGTGCCA	105.4
	R	ATTCACGTAGTCAGCTCGCA	
PYGL	F	ACTGACCCCTGCGTGTAAAG	104.2
	R	TGGAGGCAGGGTTGATCTTG	
OSBPL8	F	GCTGGACGGACATCACTTGT	98.9
	R	TGGATGTCTACCACTCGGGA	
ApA	F	GTGCCGGGAAAGTGATTTTAG	99.6
	R	CAGCCACCAGGGTCGCGA	
Act	F	GAAGAGCTACGAGCTTCCCG	102.1
	R	CATGGATACCGGCAGACTCC	
PFK2	F	AGCGCACTATCCAAACTGCT	111.6
	R	TCTGCAAAGTCCTGGGGGTA	
GAPDH	F	ATACATCAGGAACAGGGACTC	93.9
	R	GACTTATGACAACCGTGCA	
rpL10	F	TGCACGTGAGGCTGATGAAA	102.3
	R	GTGGCCACTTTGTGAAACCC	

Figure S1

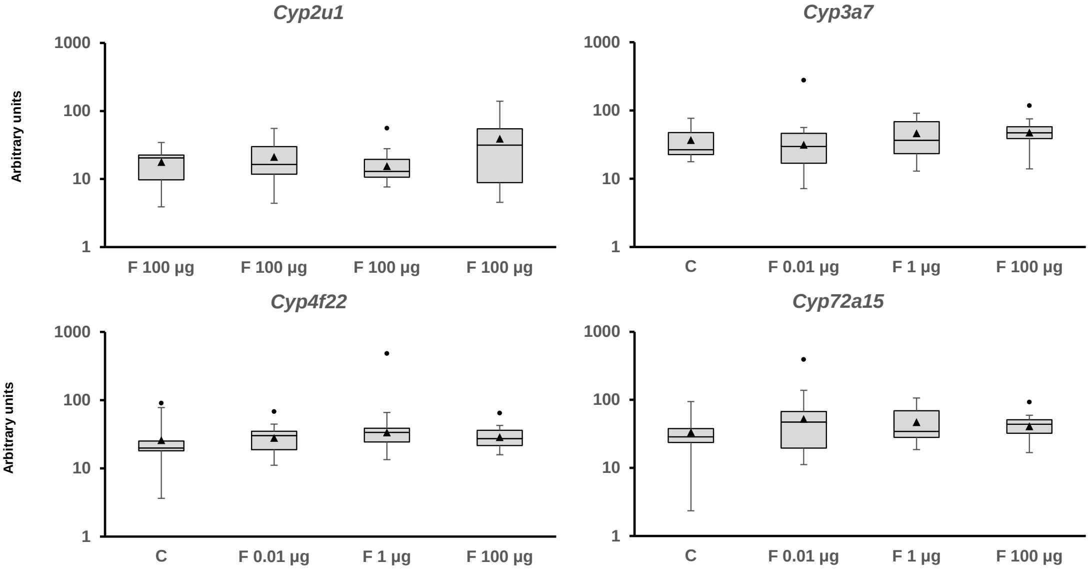

Figure S1. Transcript levels of cytochrome P450 genes (*Cyp2u1*, *Cyp3a7*, *Cyp4f22*, and *Cyp72a15*). Adult snails were exposed for one week to Fenoxycarb at 19 °C. The mRNA levels were analyzed by RT-PCR using *rpL10*, *Act*, *PFKFB2*, and *GAPDH* as reference genes. The comparison was performed with the solvent-exposed controls. Whisker boxes are shown. Each box corresponds to 12 individuals. The median is indicated by the horizontal line within the box, and the 25th and 75th percentiles are indicated by the boundaries of the box. The highest and lowest results are represented by the whiskers. The small triangle inside the box denotes the mean, and the outliers are shown (circles). No significant differences relative to the control were detected ($p < 0.05$).

Figure S2

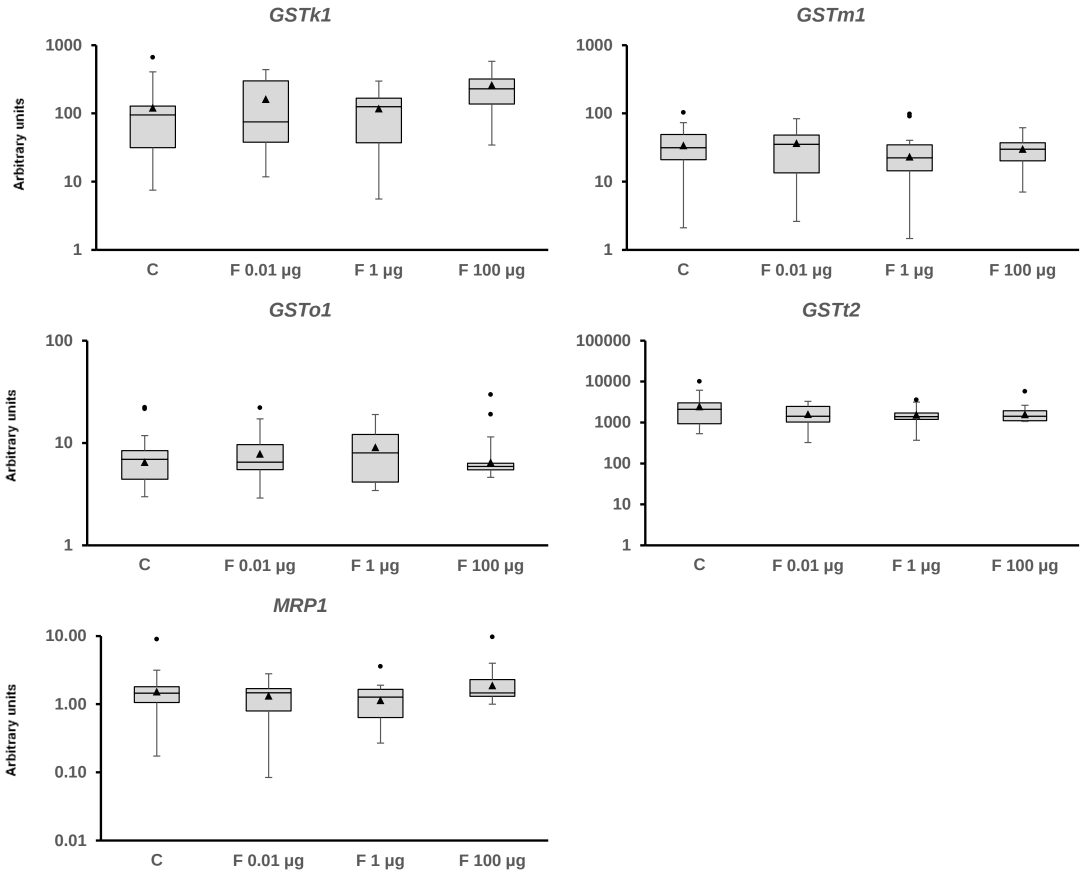

Figure S2. Transcript levels of Phase II related (*GSTk1*, *GSTm1*, *GSTo1*, and *GSTt2*) and Phase III (*multidrug resistance protein 1*) related genes in *Physella acuta* adults after *in vivo* exposure to Fenoxycarb for seven days at 19 °C. Transcriptional activity was quantified by RT-PCR using *rpL10*, *Act*, *PFKFB2*, and *GAPDH* as reference genes. The comparison was performed with the solvent-exposed controls. Whisker boxes are shown. Each box corresponds to 12 individuals. The median is indicated by the horizontal line within the box, and the 25th and 75th percentiles are indicated by the boundaries of the box. The highest and lowest results are represented by the whiskers. The small triangle inside the box denotes the mean, and the outliers are shown (circles). No significant differences were detected relative to the control condition ($p < 0.05$).

Contig1634 - Estrogen related receptor (3125 bp) – CDS: 72-1400

GGCTGGTAATTGTTCTCCTTAATTAGGAGAGCAATTCACACACTTAAACAGCAAAAATTGATTTGTTCAATAT
GGAAACCGACGACAGTAACTTAGCCTACCGTCTAGGAACTCTGTGGGCTGGTATGGATCTAGACACAGTCATT
AAATGTGAGCCAAACAGTCCTCTCGGCCTGGGACGGTGCAACCAGCCCAACCTGGACGACAGCTTCCCTCGGG
ATGTCTTCGCCTACGACCCCTCCCACGACGACTACGGGTCGGACCGTTCCTACCAGGGAGACAGTGCCGACAG
TGGGAATATATCCCCGTGTAGCTTAGACAATATCAAAATCGACTTCTGCTCCACTACGTTTAACACTGAGAAC
GGTGAAGGCACTGACGGAGCTAAGAGACTTTGTCTTGTGTGTGGTGATGTGGCCTCTGGATATCATTACGGTG
TCTCCTCTTGCGAGGCGTGTAAAGCATTTTTCAAAAGGACAATTCAAGGTAATATAGAATACTCATGTCCAGC
TAGTGGCGACTGTGAGATCACAAAACGTAGGAGGAAGGCTTGTCAGGCTTGCAGATTTCAGAAATGTCTGACT
GTTGGCATGTTGCGAGAAGGTGTCCGTCTAGATCGTGTCAGAGGTGGAAGACAAAAATACAAACGTAGTTCAG
ACTGTCAAACACATGTACAGCCCATTATACCAATGGTCATAAAGAAGTCATGTTTAGAAGTTGCAGACAATAA
AATCCTGACAATGTTGCTAGCACTAGAGTCACAGATGGAAGTATTACGGGCCAACACAGACACAGCTATGGAT
TCAGATGTGAAGTTTATGGCTGCAGCATCTGACCTCGCTGACAGGGAGTTGGTCATGACCATCAGTTGGGCCA
AGCAGGTTCCAGGTTTCTCCGCCCTGTCCCTAGTTGATCAAATGATGTTGTTACAACATTCTTGGCTGGAAAT
CCTTATGTTAAGTCTTGTCTTTCGCTCCTGCCCTTACAAAGGTCAGATCTGCTTTGCAGAGGACCTTCAGATA
TCCGAAGAAATGGTAGAAACTCTAAACTTTCCTTCAGAGCTGGATGCTCTTGTACGCAAGCTTAGTAAGAAAT
TTACGCACCTCAATGTCAGCAAAGATGAATTTGTACTCCTCAAGGCCATAACACTTTGTAATATTGATGTTGT
AGCAGAGCTTGGCGAGTGTGTAGCGCACCTACAGGACCAGCTGCAAGATGCTCTCTCAGACTGTGTCAAGTCT
GTCTATGGCCAGAACACTAGGCGTCTGGGACAGCTGTTCCTCTTGCTGCCATCCATAACACACATCAAGCTCC
TGGCCAAACAGTTCTGGTACGACATGAAGAAAGATGGCCGCGTCATGATGCATAAGCTCTTCCTGGAAATGCT
GGATGCAGACTCCTGAGATTGGCTCAACTGTAGGGAAAGCTCTTGTTGCTGCACATTTCAAAGGCTGGAATGT
ACATATTCATCATCTAAGTTTCTAGTTCCCTAGTGTAGTCATGCTGTAACCTGCCTTAGACTAATTAGCTAGC
CACAATGTTTTAACCAAGCAATATTTTTGAGGAAGAAAAAAAATTATGGACTGAAGAAATGGACAATACCACC
AGCCTTTCATCGACTACAAAACAAAAGTAATTGAAATCAATTTTTCCCAGCAAAAAAAAAAGATGGTAAGGCG
TCATTAGTCACATACTTTTGTATTGAGCCTGTGGCCAACCATCCAATGTGAAGTTTTCTCAAGAAAATGTATC
AATGAAGGCAGCCAGGGTCATCTCCCTGTGTAGGCTGTTGCTAGTGGGTGTCCTACAGCACAATCATCAAACA
GGAGATGACACTTTGTTTATTTTGGTTTTGTTTTTGAAAACCACTCCACTCAAATTATCAACGATTCAATATT
TTTAAAAGGGTTTTGTTTATACCTAAATTCAAATGATTTAAGGCATTTTGGCTAAAACACTTGCCATGCATTA
TACTTCATTTTCTGTCAATGACTTGCATCTTCAATATTTAAAGGGGGGTAACTGGTCAACCAGTTTAAATTAT
TTTATTTTGCTGTGGGAACAATTCACAATGGGTGTTCCCTGGGGAATGGCATCTCATTTCATTTGGGAAAGTA
ATTTTTTTAATCTGTTGAGAAGTATTATATTTTTGTTTTAGAAATGAATGAAAAATAACTTGTGATGATTAGA
TATCAAACCTTTTTTACGATATTTTTTAAAACAAATTATTTCAAGATTTGAACCTCAGGTCTAGTTACTGCAA
CAATGATCAAATGTTACTTGTCCACGTTTGGTGAATTCTCTAGTTGCTATGGCAACTTTTCAAATGGACTTTT
TCTATTAAAAAAAAATCTTTAAATATTACAGACCATTATTTTAAAATTTTAATCATCTAAATATGTATTTGGG
GTGGGGAGGTGGGGAAACTAAAAGATTTTATAGAGCACTCAACTAAGGTAACTTTCGCCCCTTAACACAATTT
TTTAATCTGTTGAAATGTCAGATCAATTTTGCTCACTTATTTATTTTCACCTGCATATCAAACTTAAGTTATT
TACCATTCATTTTTACCTCTACAAATATAAACCGATGAAACATGCACCTCCCTTTCATTTCTCAACTATAAAT
ATATCACCCCATGAAAGAATTAGTATTGATCTGTCAGATAATAATGGACTGTAGAGCATTCAGTGGTTTAAAC
ATTTTTATTTCCTGTAGATTCCACATTGCCCCAAGCTTCTTATGTTGTATCACTAAACTATTGTACTAGATGA
TGAATGTGCACAGATCATATTGTCCCACCCCGGATAGCCAGGTTTATAAGATTCCTTATTTATTGAATCTACC
GGCTGATACTGTACTAGTAAAACCAGTGGAGAACTAGGCCTCTTCTGCATTTGTTTACAAACATGTCATCCCA
TACTACTTGGTTAGTAAAAAAAAAAAAAAGCGTATATTATCTATGCTTAAGAGTGTAGACCTATATAGCCAGAG
AAATACATCAATTTAAGTCAACAGAAAACTCATTTTTAAGTGATTGTCAACACATGAGTAATGTAAATAAATC
ATTGTTTTAAAATTATTAAGAAAGTCATTTGAAAAACTAATTCAATAGACTTAAACTTT

Protein (443 aa)

METDDSNLAYRLGTLWAGMDLDTVIKCEPNSPLGLGRCNQPNLDDSFPRDVFAYDPSHDDYGSDRSYQGDSAD
SGNISPCSLDNIKIDFCSTTFNTENGEGTDGAKRLCLVCGDVASGYHYGVSSCEACKAFFKRTIQGNIEYSCP
ASGDCEITKRRRKACQACRFQKCLTVGMLREGVRLDRVRGGRQKYKRSSDCQTHVQPIIPMVIKKSCLEVADN
KILTMLLALESQMEVLRANTDTAMDSDVKFMAAASDLADRELVMTISWAKQVPGFSALSLVDQMMLLQHSWLE

ILMLSLVFRSCPYKGQICFAEDLQISEEMVETLNFPSELDALVRKLSKKFTHLNVSKDEFVLLKAITLCNIDV
VAELGECVAHLQDQLQDALSDCVKSVYGQNTRRLGQLFLLLPSITHIKLLAKQFWYDMKKDGRVMMHKLFLEM
LDADS

Contig8155 –NF-kappa-β inhibitor alpha-like (1611 bp) – CDS: 183-1313

CAAGAGGTAGATCTAGACTAGAGCTAGTTAAACTTTTAAGTTAGATCTAGACTAGACTTTGTAGATTCACTCT
CGCGACTTGAGGTATGGCTTTAAAAATTGTTTAAATTTCAAAAGTGAACAAACCATGATTAGAATCTAGACTA
GATCTAGATTTCTAGGTCTAGATAGTTGTTTTATAAATGGAAGAAAACTATGAATCAGGTACGGACATTTTAA
AGACAATGTGTAGTCCGATAGAAGAAAGAATAGATTCTGGATTAGGATCACTTGGTTATGTATCATCGGATAC
TAGCAACTTGTCGATAGCTGAAGAACCAGTGACTGACAATACGGCCAGTAGATCCCGGTCTGTTGAAAAAAAC
ACAGTCGACCTTAGTCATCAACTTTCTCTTATTAGTATTGATAGTTTAAAAGAAAATTGTGATTCGGGAGTTG
ATGAGAACTTCATATCGAATTTGAGAAGCCCAGATTCACAAGTTTTGGAAAACTGGTCGTCACCAAGTTTTAC
AGCGGAACAAATTGTTGAAATATTTCGAGGTGATGAAGATGGAGACAACCATTTACACTTGAGTATCATCCAT
GGCTTGCCAGAGGTGACCATGCAAATCATTGGGTTGGCACCAGACTTGGATTGGTTGAATCAGACCAATAGCC
TCTTACAAACACCCTTACACATTGCAGTCATCACAGGACAGACTTGTGTTGTTGGGAGGTTGATGGCAGCTGG
GGCAGCACTGGATGTCAGAGACCAGCTTGGGAACACTCCCCTACACAATGCTTGCAGGCTGGGCTTCACAGAC
ATTGTACGGACATTGATCACACCTGGTCATTATGAAGAAACTTTGCAGAACCAGTATGGCATTCCAAATCAGC
AGTTTCCCCAAGATTTGGAGTTAAAAAACTATGAAGGTCTGACTTGCCTCCACCTTGCTGCTATTGGAGAACA
TGTGGATGTGATGCAGCTGTTACTGGCTGCAGGAGCAGACGTCAATCAGCCAGAGGGCAAGAGTGGACGCTCT
GTCTTACACTTGGCTGCAGAGTGGGGAAACCTCAACATGCTGCGCTTTTTACTTTCATTTCAAGAGACCCACA
TTGATGCACAAACTTATGCAGGACTAACACCTATTCTGCTGGCAGTGGGGCGTAAACATGAGGACATTGTGAA
GGAGCTTTTCAAACACGGAGCACTTTTAGAGAGACTATCATTGAGTGATGATTCTGACATTTCAGATGATGAA
ATGAATGGAGATGCATCAGAATACAGAACATTTGGACTCTACCAACCAAAGTTGTGCCAGAATACATGTGGGT
AGCCCAGCTTTAGCAGTCGGCTGGGATCCAGATGAACAGGACTACTCTGGAGGTTCAATGTGAAGCTGGCAGA
TGACCAGTACCAAATGAGACACCATCATGTTGGCATCCCAAGATGTCACTCAGTAAATAAAAAGGTGTTGGCC
TGTTCTCAACCATTTATTTAGCACCTACTGTAGAGCAAGCAAGCAACTGTTCTCATTGTATATATGTACAGAG
GGCTCTGGTGTTTGTTGTAGAGGACAGTCCTAAGGCTTGAGCTCTTGTAAGCAGTCTAGAGAAATAGATACTA
AAAAT

Protein (377 aa)

MEENYESGTDILKTMCSPIEERIDSGLGSLGYVSSDTSNLSIAEEPVTDNTASRSRSVEKNTVDLSHQLSLIS
IDSLKENCDSGVDENFISNLRSPDSQVLENWSSPSFTAEQIVEIFRGDEDGDNHLHLSIIHGLPEVTMQIIGL
APDLDWLNQTNSLLQTPLHIAVITGQTCVVGRLMAAGAALDVRDQLGNTPLHNACRLGFTDIVRTLITPGHYE
ETLQNQYGIPNQQFPQDLELKNYEGLTCLHLAAIGEHVDVMQLLLAAGADVNQPEGKSGRSVLHLAAEWGNLN
MLRFLLSFQETHIDAQTYAGLTPILLAVGRKHEDIVKELFKHGALLERLSLSDDSDISDDEMNGDASEYRTFG
LYQPKLCQNTCG

Contig11036 - DNA repair protein XRCC3 (1274 bp) – CDS: 107-1141

ATTACATTAAGCCCGATATGAGGCCTAGATCTAGGTCTTCTAATACATTTTTAAAAAAAACTATTAATCTGTT
ATGAAAACACTAGTCTAGCTGAAAGCTTCAGCCATGCATTACCAAAACGAAGAAATATATAAATTTAATCTTG
ATTTAAACCCTAAAATTATTACTGCCCTAAAGAAAGCCATGCTAACAGACTGCAGATTGATACTAAGTTTATC
AAATCCAGACATCCAGCGCCTCACTAGTCTTTCACTTACAGAAGTCTCTGCACTAAAAGACTCATTAGCAGAA
AAGTATATACTTAAACCATTCAATGTTTTAGATATTCTCAACAACAAAGCTGACAGTTCCTTGCAACATTGGA
AGCTGTCATCATTTTGCAAAATAATTGATATATCTTTGAGAGGTGGTTTTCTATCTGGGTTAATTACAGAAAT
TTCTGGTGAAAGTGCATGTGGAAAAACACAACTTTGTCTTCAGCTATGTATAAGCGTTCAGTTGAACAACGAA
CATCCTGGAGGGGCTGTTTACATTTGTACGGAAGATGCATTTCCAAGCAAGCGTCTCCAGCAATTGATTCAGT
GCCACTCTTCTAAATTACCGTCTTGTAACTTGGGAGATAATATATTTATAGAACATGTAGCAGACTTTGAAAC
GCTGGAGTTTTGTGTAAATAAGAAATTACCTACCCTCCTTAAAAGAGGATTGGTGCAACTGATCGTGATAGAT
TCAGTGGCTGCTCTATTTCGCTGTCACTATGATCACACACAGACTGTAGAAAGGGCAAAACACCTGACCAAGT
TTGCGTCTGTATTACGGAACCTTGCATTTCAATACAATATCCCTGTGATTTGTGTGAATCAGGTTTCTGCTAA
TTTGAGTCCACCTTCTGGACAGAGCTTAGTCATTCCTGCATTAGGTCTAACATGGTCCAATCAAATCACAAGC
AGAATAATGCTTTGCAAACCATATTTTACTCAAGGTTTAGAACATCTAGACACAAAGTCTTTGAAGAACAATG

AACCAACACATAGAAAACTCAGAGTAATATTTGCTCCACATGCACCAGAAAGTGAAATTGATGTGTATATTGA
TAATACTGGAATTCATGGAATATCAAGAAAAGTTGTTGAAAAGATGTAGTGTTCCAAATGTAAATTGATTTGT
TCTAGAGTCTTATGACTATTTTAATTCTAAATATTGGTGGCTGAGAATGTTTAAATATCATTAAATGTACGTA
AGTACATGTAGTGACTTGAAATAAAAAAGTGT

Protein (345 aa)

MHYQNEEIYKFNLDLNPKIITALKKAMLTDCRLILSLSNPDIQRLTSLSLTEVSALKDSLAEKYILKPFNVLD
ILNNKADSSLQHWKLSSFCKIIDISLRGGFLSGLITEISGESACGKTQLCLQLCISVQLNNEHPGGAVYICTE
DAFPSKRLQQLIQCHSSKLPSCNLGDNIFIEHVADFETLEFCVNKKLPTLLKRGLVQLIVIDSVAALFRCHYD
HTQTVERAKHLTKFASVLRNLAFQYNIPVICVNQVSANLSPPSGQSLVIPALGLTWSNQITSRIMLCKPYFTQ
GLEHLDTKSLKNNEPTHRKLRVIFAPHAPESEIDVYIDNTGIHGISRKVVEKM
